# Tea nanoparticle, a safe and biocompatible nanocarrier, greatly potentiates the anticancer activity of doxorubicin

**DOI:** 10.18632/oncotarget.6711

**Published:** 2015-12-22

**Authors:** Yi-Jun Wang, Yujian Huang, Nagaraju Anreddy, Guan-Nan Zhang, Yun-Kai Zhang, Meina Xie, Derrick Lin, Dong-Hua Yang, Mingjun Zhang, Zhe-Sheng Chen

**Affiliations:** ^1^ Department of Pharmaceutical Sciences, College of Pharmacy and Health Sciences, St. John's University, Queens, NY 11439, USA; ^2^ Department of Biomedical Engineering, College of Engineering, The Ohio State University, Columbus, OH 43210, USA

**Keywords:** tea nanoparticle, tumor xenograft, multidrug resistance, drug delivery, biocompatibility

## Abstract

An infusion-dialysis based procedure has been developed as an approach to isolate organic nanoparticles from green tea. Tea nanoparticle (TNP) can effectively load doxorubicin (DOX) via electrostatic and hydrophobic interactions. We established an ABCB1 overexpressing tumor xenograft mouse model to investigate whether TNP can effectively deliver DOX into tumors and bypass the efflux function of the ABCB1 transporter, thereby increasing the intratumoral accumulation of DOX and potentiating the anticancer activity of DOX. MTT assays suggested that DOX-TNP showed higher cytotoxicity toward CCD-18Co, SW620 and SW620/Ad300 cells than DOX. Animal study revealed that DOX-TNP resulted in greater inhibitory effects on the growth of SW620 and SW620/Ad300 tumors than DOX. In pharmacokinetics study, DOX-TNP greatly increased the SW620 and SW620/Ad300 intratumoral concentrations of DOX. But DOX-TNP had no effect on the plasma concentrations of DOX. Furthermore, TNP is a safe nanocarrier with excellent biocompatibility and minimal toxicity. *Ex vivo* IHC analysis of SW620 and SW620/Ad300 tumor sections revealed evidence of prominent antitumor activity of DOX-TNP. In conclusion, our findings suggested that natural nanomaterials could be useful in combating multidrug resistance (MDR) in cancer cells and potentiating the anticancer activity of chemotherapeutic agents in cancer treatment.

## INTRODUCTION

Cancer is well defined as a disease in which abnormal cells divide without control and invade nearby tissues, and is often clinically referred to as tumor or malignant neoplasm. Cancer has been ranked as the second most primary cause of death after cardiovascular diseases in the world [1]. Cancer is comprised of approximately two hundred potent, heterogeneous diseases that originate in specific organs such as the lung, breast, colorectum and prostate, rather than a single disease [2]. Cancer therapy usually includes surgery, radiation therapy, chemotherapy and combination therapy. As a first-line treatment for various cancers, chemotherapy uses drugs with different chemical structures and mechanisms of action. Chemotherapeutic drugs combat cancer by disturbing cell proliferation and targeting the fast-dividing cancer cells [3]. However, long-term cancer chemotherapy often results in the development of resistance to anticancer drugs and attenuates the efficacy of cancer treatment, leading to higher chances of “cancer relapse” [4].

Multidrug resistance (MDR) appears to be the leading blockade in chemotherapy. It is a phenomenon in which cancer cells become resistant to drugs with different chemical structures and mechanisms of action [5]. Interestingly, the mechanisms of MDR are very complicated and comprised of alterations in the permeability of lipid bilayer membranes, suppression of apoptosis, upregulated DNA repair of cancer cells, inactivation or detoxification of drugs, changes in the number of membrane receptors or transporters involved in accumulating or effluxing drugs from cells [6]. The overexpression of a family of specific transmembrane, energy-dependent transporters known as ATP-binding cassette (ABC) transporters is one of the primary mechanisms that cause MDR in cancer cells [7]. ABCB1 or P-gp (Phospho-glycoprotein) is the first ATP-dependent system to be discovered [8, 9]. As an apical plasma membrane transporter, ABCB1 is ubiquitously expressed in kidneys, intestines, placenta, liver, adrenal glands and blood-brain barrier (BBB) cells, where it normally functions to extrude certain xenobiotics and protect cells from toxicants [10, 11]. It has been reported that overexpression of ABCB1 plays an important role in eliciting MDR in cancer chemotherapy and displays high resistance to a wide variety of substrate anticancer drugs, such as anthracyclines [doxorubicin (DOX), daunorubicin], vinca alkaloids (vinblastine, vincristine), taxanes (paclitaxel, docetaxel), epipodophyllotoxins (etoposide, teniposide), imatinib mesylate (STI-571; gleevec) [12, 13], antibiotics (dactinomycin and actinomycin D) [14], some HMG coenzyme A (statins), antihistaminics, antiarrhythmics, steroid hormones, calcium channel blockers and HIV protease inhibitors [11]. Furthermore, ABCB1 has been found to be overexpressed in different cancers, such as gastrointestinal stromal tumor (GIST), non-small cell lung cancer (NSCLC), fallopian tube, ovarian and thyroid cancer [15–19].

In recent years, much attention has been paid to the development of natural nanomaterials, such as viruses [20], lipoproteins [21], diatoms nano-biosilica [22], ivy nanoparticles [23, 24], and fungal nanoparticles [25]. This is because natural nanomaterials have excellent biocompatibility and less toxicity as compared to chemically synthesized nanomaterials. Natural nanomaterials also have characteristics desired for medicinal applications [26–28]. In traditional Chinese medicine, tea is a healthy beverage. Tea is loaded with antioxidants and nutrients that have powerful effects on the human body. It is reported that tea can improve brain function, promote fat loss, lower the risk of cardiovascular disease, cancer and type II diabetes, as well as with many other incredible health benefits [29, 30]. It has also been reported that tea is associated with many therapeutic effects, including anti-hypertension [31], anti-blood coagulation [32], HIV treatment [33], oxidative damage repair [34, 35] and cancer prevention and treatment [36, 37]. Chinese people have been drinking tea for thousands of years. It is well known that certain tea derived compounds have antineoplastic capability. However, little is known about the effects of tea derived compounds on MDR.

**Figure 1:**
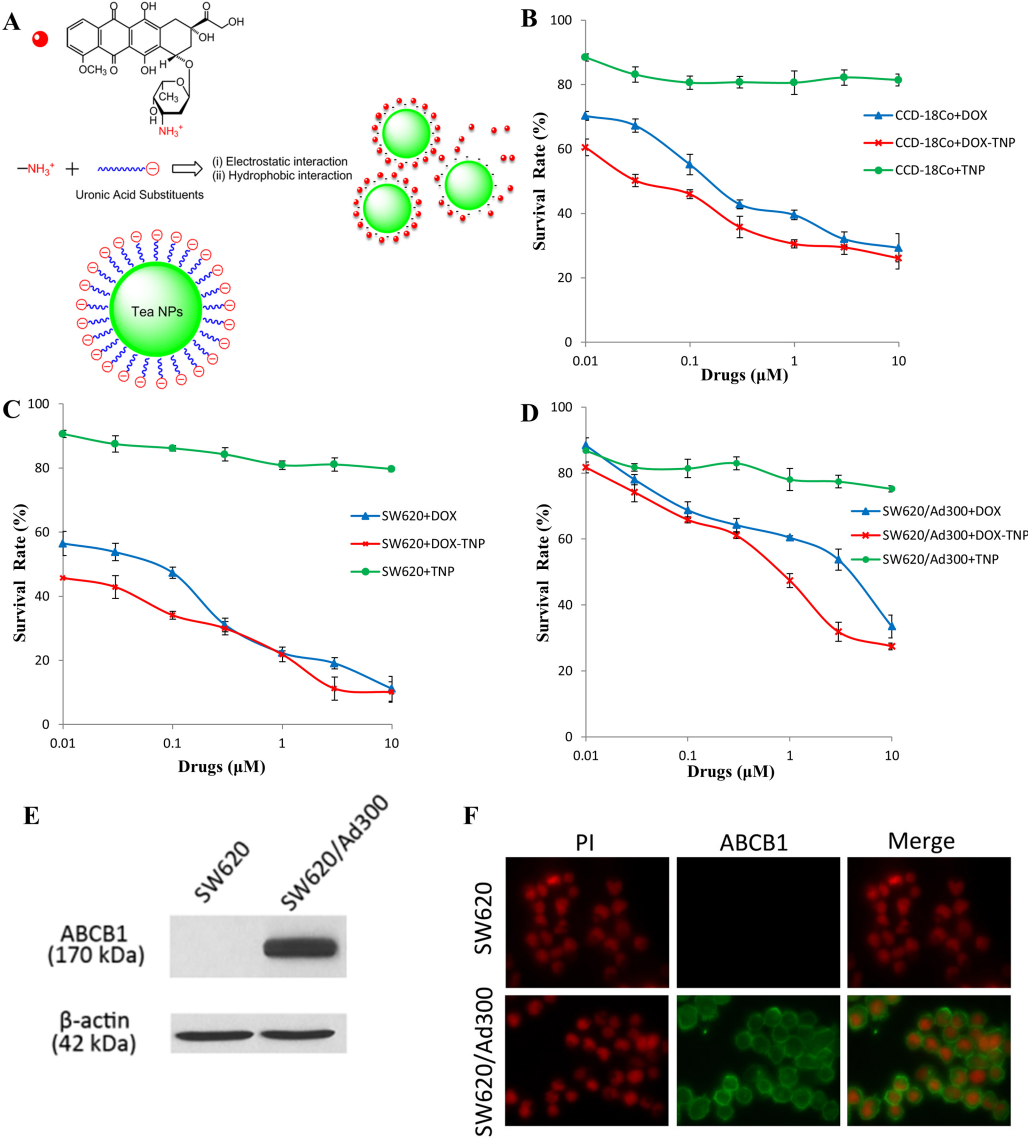
(**A**) Concentration-response curves of human normal colon fibroblast cell line CCD-18Co treated with doxorubicin (DOX), DOX-loaded Tea nanoparticles (DOX-TNP) and TNP. (**B**) Concentration-response curves of human colon cancer cell line SW620 treated with DOX, DOX-TNP and TNP. (**C**) Concentration-response curves of SW620/Ad300 cells treated with DOX, DOX-TNP and TNP. (**D**) Schematic drawing of the electrostatic and hydrophobic conjugation of TNP and DOX. Each cell line was incubated with different concentrations of DOX, DOX-TNP or TNP for 72 h. Cell survival rate was determined by the MTT assay. Points with error bars represent the mean ± RSD. Each above figure is a representative of three independent experiments, each done in triplicate. (**E**) Immunofluorescence assay showing the expression level and localization of ABCB1 in SW620 and SW620/Ad300 cells. (**F**) Western blot analysis showing the expression level of ABCB1 in SW620 and SW620/Ad300 cells.

In a previous study, we developed an infusion-dialysis based procedure for isolating organic nanoparticles from green tea. It has been revealed that isolated tea nanoparticle (TNP) is spherical with diameters of 100–300 nm and have a zeta potential of −26.52 mV at pH 7.0. Chemical analysis indicated that (−) Epigallocatechin gallate, caffeine, and theobromine were not found in TNP [38]. Moreover, the TNP had an immunostimulatory effect by increasing the secretion of numerous cytokines (IL-6, TNF-α, and G-CSF) as well as chemokines (RANTES, IP-10, MDC) from mouse macrophages RAW264.7. Importantly, TNP is capable of loading DOX effectively through electrostatic and hydrophobic interactions (Figure 1A). DOX-loaded Tea nanoparticle (DOX-TNP) promoted the intracellular uptake of DOX and showed higher cytotoxicity in A549 human lung cancer and MCF-7 breast cancer cells. Furthermore, DOX-TNP greatly enhanced DOX uptake and cytotoxicity in MCF-7/ADR multidrug resistant breast cancer cells [38]. These findings have triggered our curiosity to explore if these *in vitro* results could be translated into the *in vivo* animal model. In the present study, we investigated the potential of TNP as a multifunctional nanocarrier for chemotherapy in an ABCB1 overexpressing tumor xenograft mouse model.

## RESULTS

### Cytotoxic effect of DOX-TNP

In order to investigate the cytotoxicity of DOX, DOX-TNP and TNP on different human normal and cancer cell lines, MTT assays were performed using a human normal colon fibroblast cell line CCD-18Co, a human colon cancer cell line SW620, and its doxorubicin-selected ABCB1 overexpressing subline SW620/Ad300. As shown in Table 1, the IC_50_ values of DOX in these three cell lines were 0.190 μM, 0.077 μM, and 4.430 μM, respectively. Furthermore, the IC_50_ values of DOX-TNP in these three cell lines were 0.043 μM, 0.009 μM, and 0.852 μM, respectively. Importantly, we found that DOX-TNP showed significantly higher cytotoxicity than DOX in all three cell lines (Figure 1B, 1C and 1D). SW620 cells were 4.8-time more susceptible to DOX-TNP than CCD-18Co, suggesting that DOX-TNP had good anticancer selectivity with higher cytotoxicity to colon cancer cells than normal colon fibroblast cells. Furthermore, the IC_50_ values of TNP were greater than 300 M for all three cell lines, indicating that the TNP had such low cytotoxicity and excellent biocompatibility (Table 1).

**Table 1:** The cytotoxic effect of doxorubicin (DOX), DOX-loaded Tea nanoparticles (DOX-TNP) and TNP on CCD-18Co, SW620 and SW620/Ad300 cell lines

Treatment	CCD-18Co		SW620		SW620/Ad300	
	IC_50_ ± SD^a^ (μM)	CR^b^	IC_50_ ± SD (μM)	CR	IC_50_ ± SD (μM)	CR
DOX	0.190 ± 0.015	[21.1]	0.077 ± 0.006	[8.6]	4.430 ± 0.190	[492.2]
DOX-TNP	0.043 ± 0.010	[4.8]	0.009 ± 0.001	[1.0]	0.852 ± 0.055	[94.7]
TNP	> 300 μM		> 300 μM		> 300 μM	

a IC_50_: concentration that inhibited cell survival by 50% (means ± SD).

b CR: cytotoxicity ratio was the value of IC_50_ value for DOX or DOX-loaded TNP on CCD-18Co, SW620 or SW620/Ad300 cells was divided by IC_50_ value for DOX-loaded TNP on SW620 cells.

Values in table are representative of at least three independent experiments performed in triplicate.

### ABCB1 expression and localization in SW620 and SW620/Ad300 cells

To determine if ABCB1 is expressed in SW620 and SW620/Ad300 cells, Western blot analysis was performed. The result indicated a band with a molecular weight of approximately 170-kDa in the SW620/Ad300 cell lysates, suggesting the presence of ABCB1 protein. In contrast, this band was not present in parental SW620, indicating the absence of ABCB1 protein (Figure 1E). The immunofluorescence assays were performed to examine the localization of ABCB1 in SW620 and SW620/Ad300 cells. As shown in Figure 1F, the ABCB1 protein was highly expressed in the cell membranes of SW620/Ad300 cells and but not in the SW620 cells, which was consistent with the Western blot analysis. This result suggested that ABCB1 was induced in MDR cancer cells.

### TNP significantly potentiates the anticancer activity of DOX in the ABCB1 overexpressing tumor xenograft model

**Figure 2:**
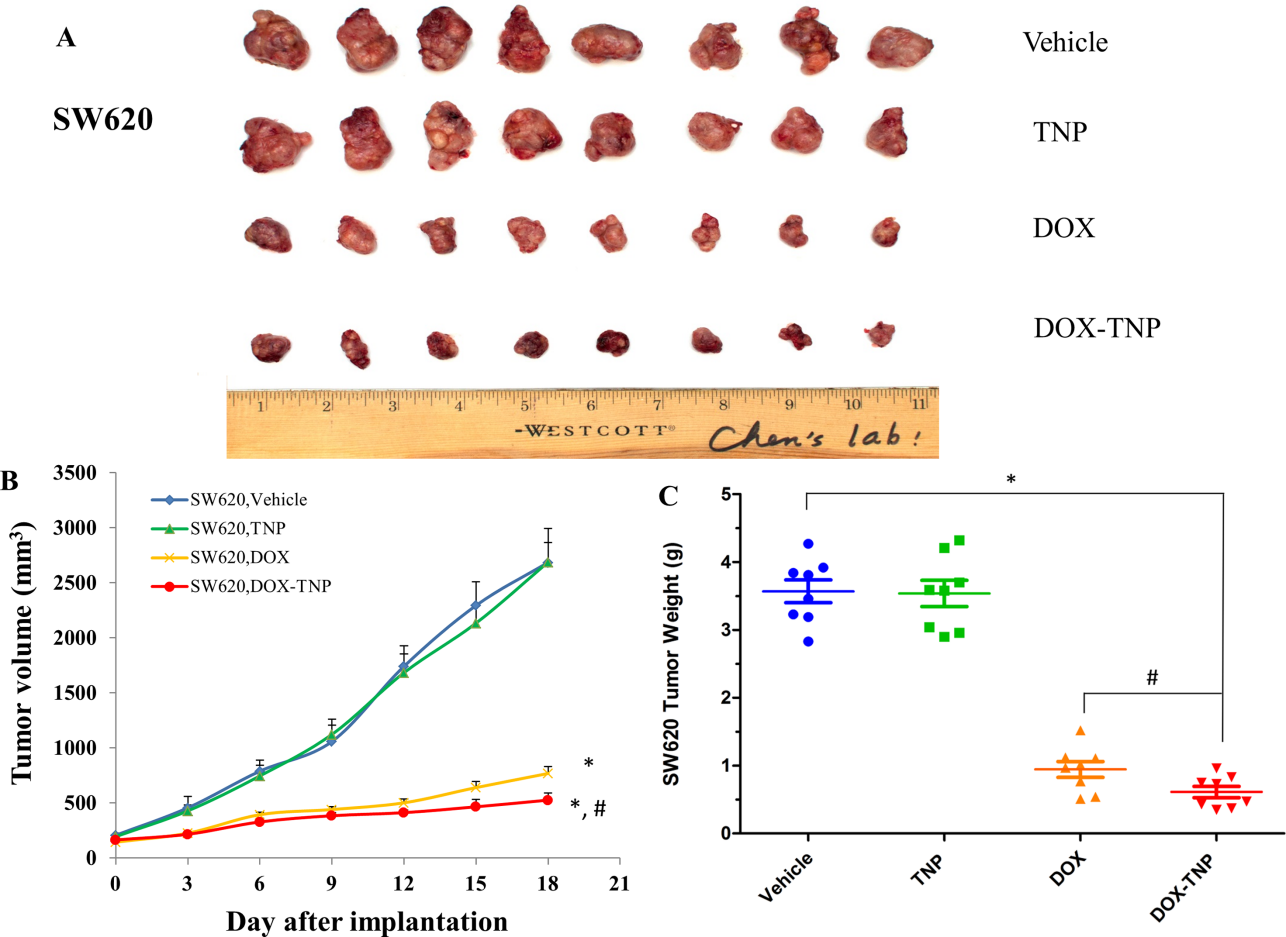
The effect of DOX, TNP and DOX-TNP on the growth of SW620 tumors in nude athymic mice (**A**) The images of excised SW620 tumors implanted subcutaneously in athymic NCR nude mice (*n* = 8) that were treated with vehicle, TNP, DOX and DOX-TNP, at the end of the 18-day treatment period. (**B**) The changes in tumor volume over time following the implantation. Data points represent the mean tumor volume for each treatment group (*n* = 8). Error bars, SEM. *P < 0.01 versus the vehicle group; ^#^*P* < 0.01 versus the DOX group. (**C**) The mean weight (*n* = 8) of the excised SW620 tumors from the mice treated with vehicle, TNP, DOX and DOX-TNP, at the end of the 18-day treatment period. Error bars, SEM. *P < 0.01 versus vehicle group; ^#^*P* < 0.01 versus the DOX group.

To investigate the efficacy of DOX, TNP and DOX-TNP in cancer treatment, tumor xenograft mouse models were used. Mice with SW620 and SW620/Ad300 tumors were administered with 2.00 mg/kg DOX, 0.71 mg/kg TNP and DOX-TNP. The change of SW620 tumor size after treatment was shown in Figure 2A and 2B showed that the tumor volumes of SW620 tumors after the 18-day treatment of vehicle, TNP, DOX and DOX-TNP were 2685.8 mm^3^, 2687.9 mm^3^, 768.4 mm^3^ and 526.5 mm^3^, respectively. The tumor weights of SW620 tumors after the 18-day treatment of vehicle, TNP, DOX and DOX-TNP were 3.57 g, 3.54 g, 0.94 g and 0.61 g, respectively (Figure 2C). Interestingly, it was found that DOX-TNP had more potent inhibitory effect than DOX on the growth of SW620 tumors over an 18-day treatment period.

**Figure 3:**
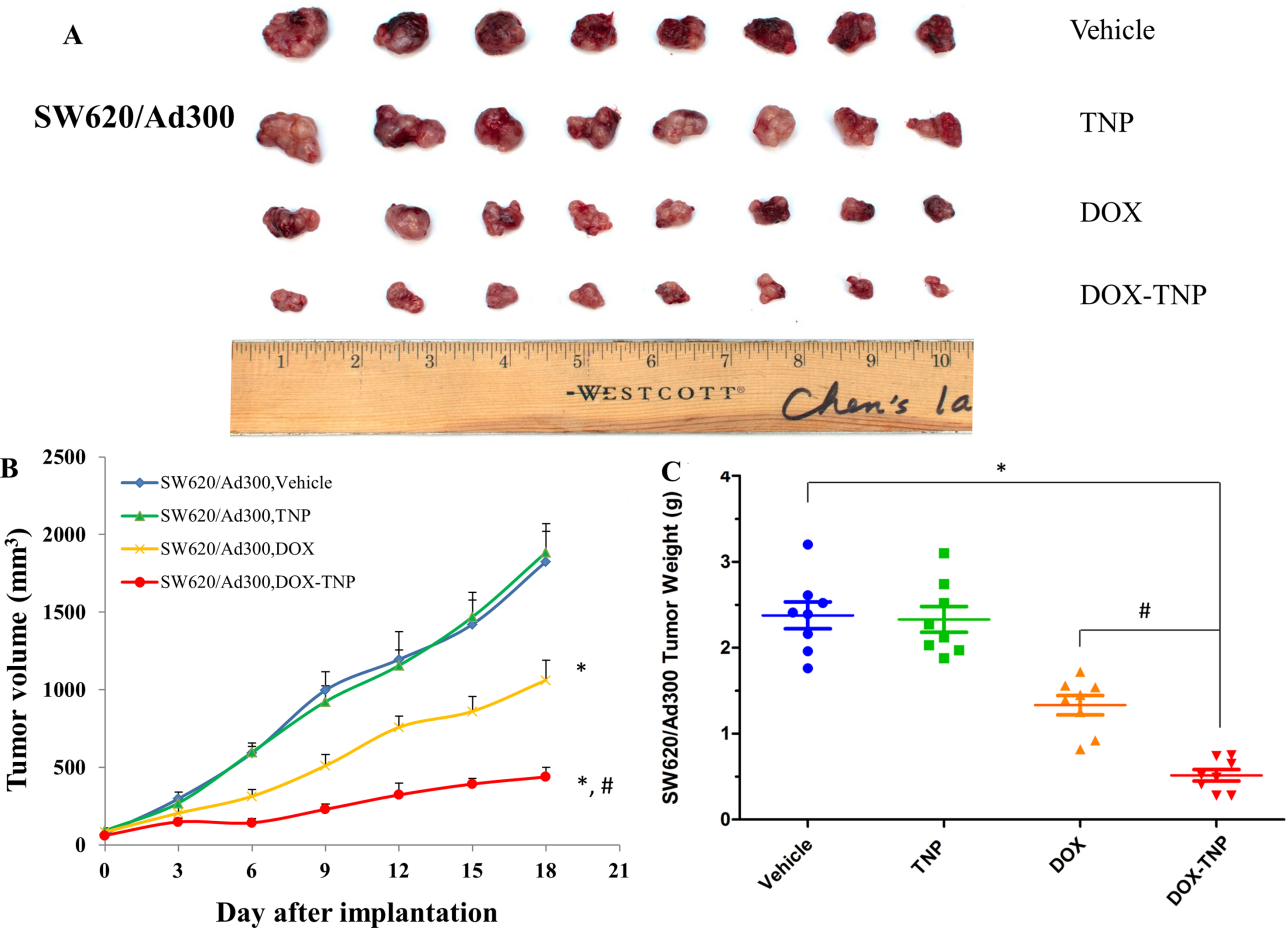
The effect of DOX, TNP and DOX-TNP on the growth of SW620/Ad300 tumors in nude athymic mice (**A**) The images of excised SW620/Ad300 tumors implanted subcutaneously in athymic NCR nude mice (*n* = 8) that were treated with vehicle, TNP, DOX and DOX-TNP, at the end of the 18-day treatment period. (**B**) The changes in tumor volume over time following the implantation. Data points represent the mean tumor volume for each treatment group (*n* = 8). Error bars, SEM. *P < 0.01 versus the vehicle group; ^#^*P* < 0.01 versus the DOX group. (**C**) The mean weight (*n* = 8) of the excised SW620/Ad300 tumors from the mice treated with vehicle, TNP, DOX and DOX-TNP, at the end of the 18-day treatment period. Error bars, SEM. *P < 0.01 versus vehicle group; ^#^*P* < 0.01 versus the DOX group.

The change of SW620/Ad300 tumor size after treatment was shown in Figure 3A and 3B showed that the tumor volumes of SW620/Ad300 tumors after the 18-day treatment of vehicle, TNP, DOX and DOX-TNP were 1826.2 mm^3^, 1885.9 mm^3^, 1062.1 mm^3^ and 438.4 mm^3^, respectively. The tumor weights of SW620/Ad300 tumors after the 18-day treatment of vehicle, TNP, DOX and DOX-TNP were 2.38 g, 2.33 g, 1.33 g and 0.51 g, respectively (Figure 3C). These results implied that TNP significantly potentiated the inhibitory effect of DOX on the growth of SW620/Ad300 tumors over an 18-day treatment period. These findings suggest that DOX-TNP has a greater inhibitory effect on the growth of SW620 and SW/Ad300 tumors than DOX alone, which is consistent with the previous *in vitro* results.

### Biocompatibility and toxicity of TNP and DOX-TNP in the tumor xenograft mouse model

**Figure 4:**
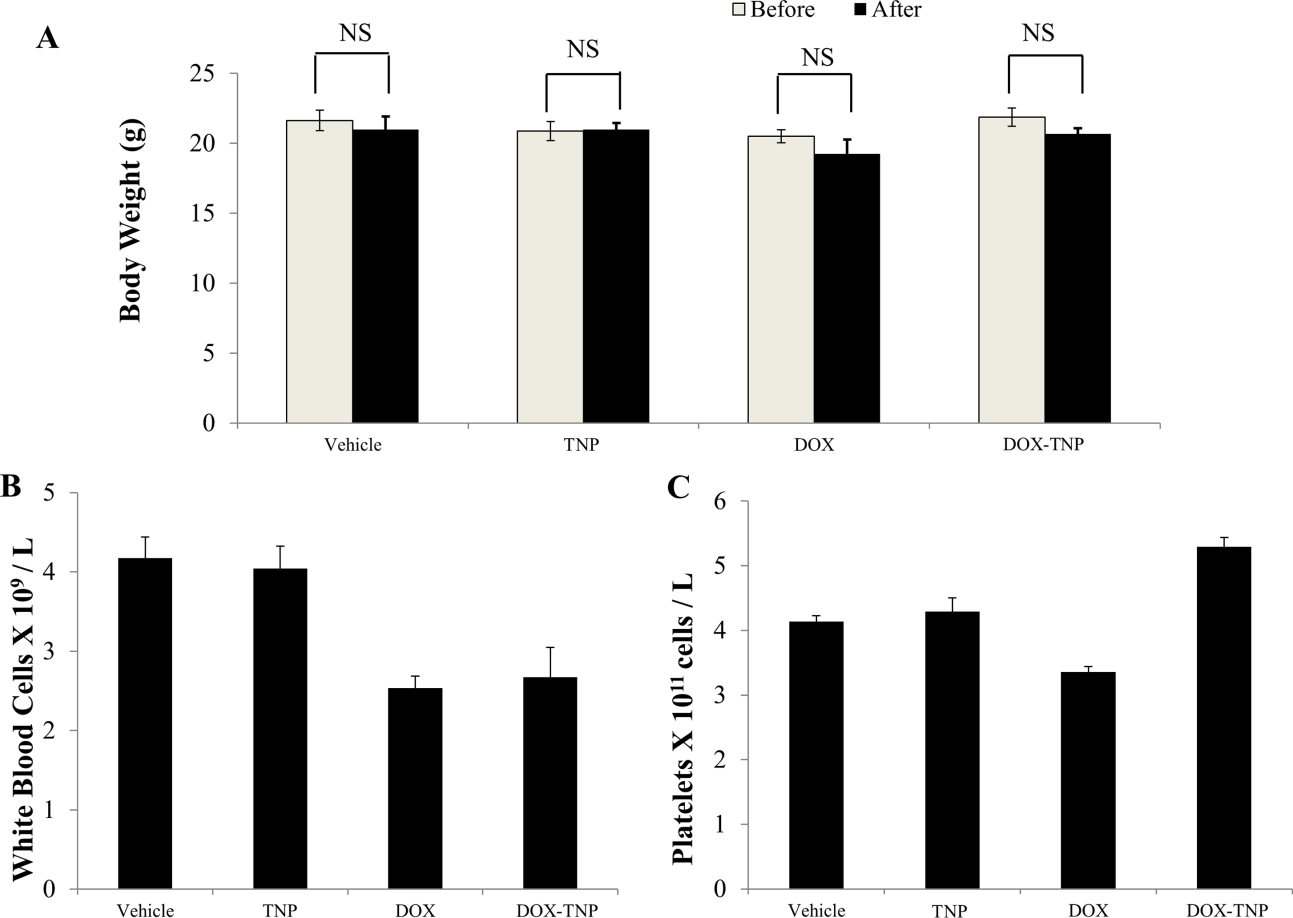
The effect of DOX, TNP and DOX-TNP on the body weight, white blood cells and platelets in nude athymic mice (**A**) The changes in mean body weight of mice (*n* = 8) before and after the treatment. NS, not statistically significant (*P* > 0.05). (**B**) The changes in mean white blood cells in nude mice (*n* = 8) at the end of the 18-day treatment period. (**C**) The changes in mean platelets in nude mice (*n* = 8) at the end of the 18-day treatment period.

Before treatment, the average body weights (*n* = 8) of the vehicle, TNP, DOX and DOX-TNP groups were 21.6 g, 20.9 g, 20.5 g and 21.8 g, respectively. The body weights of mice after 18-day treatment were 21.0 g, 21.0 g, 19.3 g and 20.7 g. No apparent weight loss was observed among the four treatment groups (Figure 4A). Hence administration of DOX, TNP and DOX-TNP did not produce any visible toxicity or phenotypic changes in mice. Since myelosuppression (neutropenia and thrombocytopenia) are the common adverse effects of DOX, we conducted blood smear tests to investigate the number of white blood cells (WBC) and platelets in mice. It has been reported that the normal range of WBC in mice are 2.32 × 10^9^ ~ 8.38 × 10^9^ WBC/L [39]. The mean numbers of WBC in mice treated with vehicle, TNP, DOX and DOX-TNP, were 4.17 × 10^9^, 4.04 × 10^9^, 2.53 × 10^9^ and 2.68 × 10^9^ cells/L, respectively (Figure 4B). The normal range of platelets in mice is 0.7 × 10^11^ ~ 12.0 × 10^11^ platelets/L [39]. Figure 4C indicated that the mean numbers of platelets in mice treated with vehicle, TNP, DOX and DOX-TNP, were 4.13 × 10^11^, 4.29 × 10^11^, 3.36 × 10^11^ and 5.29 × 10^11^ cells/L. The mean numbers of WBC and platelets in the four treatment groups were all within the normal range, suggesting that TNP and DOX-TNP did not cause neutropenia or thrombocytopenia in mice.

**Figure 5:**
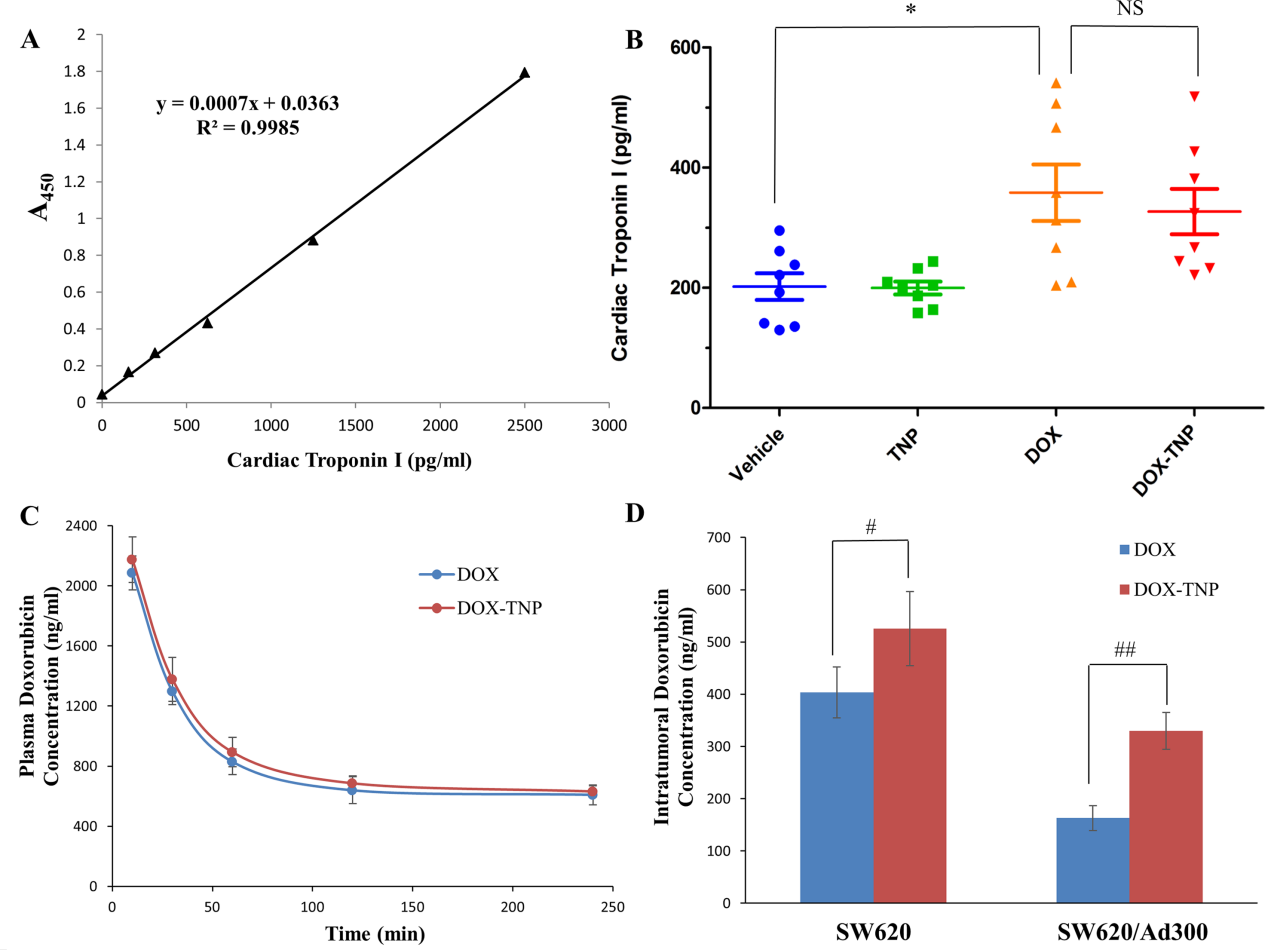
(**A**) The effect of DOX, TNP and DOX-TNP on the levels of cardiac troponin I in nude athymic mice. The standard curve illustrating the relationship between the absorbance value (A_450_) and the levels of cardiac troponin I (pg/ml). (**B**) The changes in mean levels of cardiac troponin I in nude mice (*n* = 8) at the end of the 18-day treatment period. *P < 0.05 versus the vehicle group; NS, not statistically significant (*P* > 0.05). (**C**) Plasma doxorubicin concentrations in nude athymic mice at 10, 30, 60, 120, 240 min following administration of DOX or DOX-TNP (*n* = 8). (**D**) Intratumoral doxorubicin concentrations in SW620 (*n* = 8) and SW620/Ad300 tumors (*n* = 8) after 240 min following administration of DOX or DOX-TNP. Columns and error bars represent mean ± SEM. ^#^*P* < 0.05 versus the DOX group; ^##^*P* < 0.01 versus the DOX goup.

It is well known that the most dangerous side effect of DOX is cardiomyopathy, leading to congestive heart failure [40]. Cardiac troponin-I (cTnI) is often used as a marker to indicate the damage of cardiac muscle [41]. As shown in Figure 5B, the average levels of cTnI in mice treated with vehicle, TNP, DOX and DOX-TNP, were 201.85 pg/ml, 199.71 pg/ml, 358.28 pg/ml and 351.85 pg/ml, respectively. Importantly, the cTnI levels of the TNP group were similar to those of the vehicle group, indicating that TNP had no cardiotoxicity. The cTnI levels of DOX-TNP group were slightly lower than those of DOX group, which had moderate cardiotoxicity. In addition, compared with DOX, the slight reduction of cTnI in DOX-TNP group was not statistically significant, indicating that TNP alone has no cardiotoxicity effect in mice. In conclusion, TNP is safe nanocarrier with excellent biocompatibility and minimal toxicity.

### TNP significantly enhanced DOX concentrations in tumors but not in plasma of the ABCB1 overexpressing tumor xenograft model

In a separate study, we measured the plasma and intratumoral concentrations of DOX in animals pretreated with vehicle, TNP, DOX and DOX-TNP. The pharmacokinetic data showed that DOX-TNP significantly increased the SW620 intratumoral concentration of DOX (525.5 ± 70.7 ng/ml) as compared to DOX alone (403.6 ± 48.8 ng/ml, *p* < 0.05) up to 240 min after administration (Figure 5D). Furthermore, DOX-TNP also significantly enhanced the SW620/Ad300 intratumoral concentration of DOX (329.9 ± 35.4 ng/ml) as compared to DOX alone (162.9 ± 23.9 ng/ml, *p* < 0.01) up to 240 min after administration (Figure 5D). However, DOX-TNP did not significantly affect plasma levels of DOX (Figure 5C) after 240 min following administration. These data implies that TNP-induced increment in the efficacy of DOX in both SW620 and SW620/Ad300 tumors is partially due to its better drug delivery of DOX into tumors, thereby increasing the intratumoral accumulation of DOX.

### *Ex vivo* immunohistochemistry (IHC) analysis of SW620 and SW620/Ad300 tumor sections

**Figure 6:**
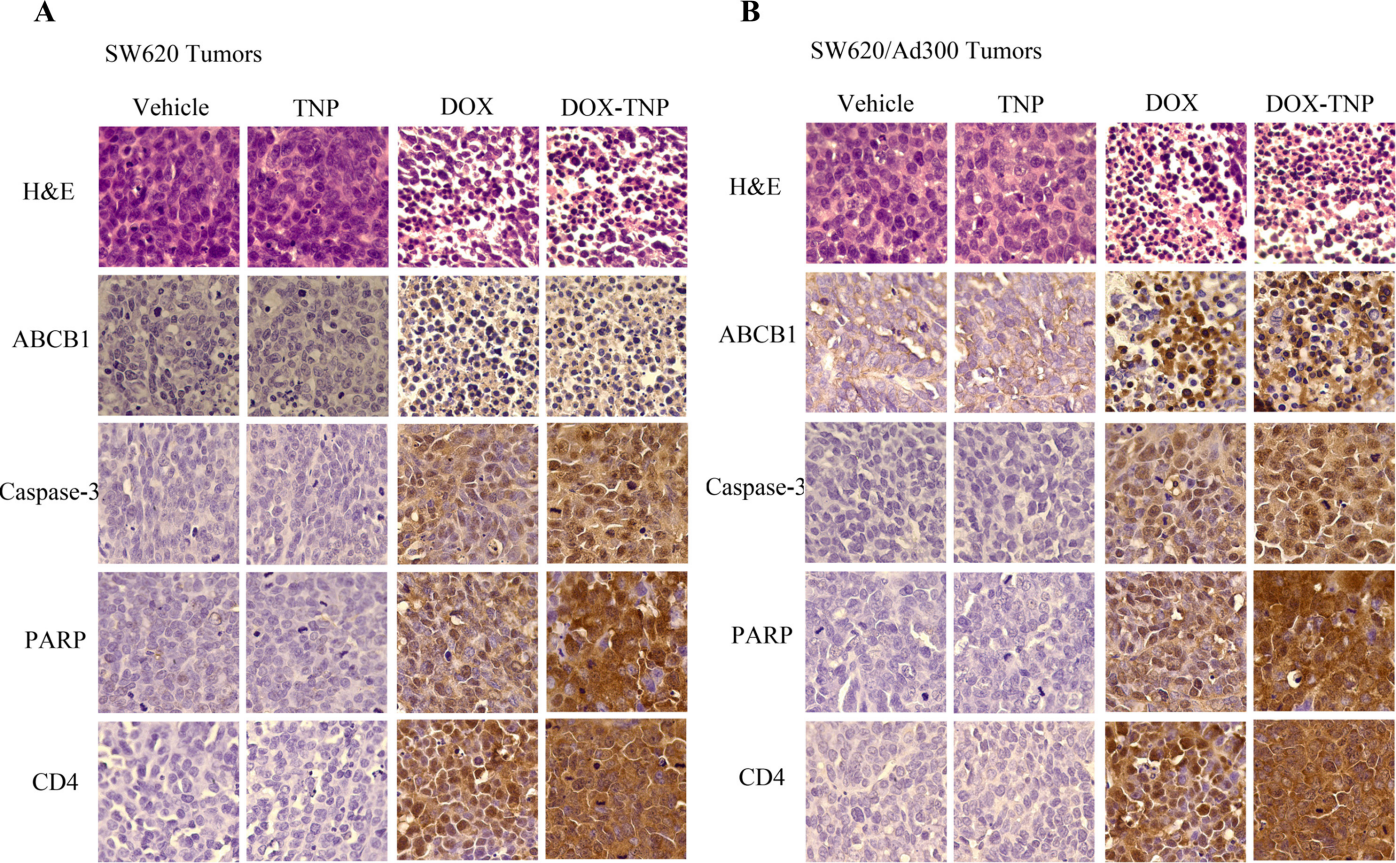
*Ex vivo* immunohistochemistry (IHC) analysis of SW620 tumor sections (A) and SW620/Ad300 tumor sections (B) In H & E staining, nuclei are stained blue, and extracellular matrix and cytoplasm are stained red. In ABCB1, Caspase-3, PARP and CD4 staining, nuclei are stained blue, and ABCB1, Caspase-3, PARP and CD4 are stained brown.

IHC analysis (H & E, ABCB1, Caspase-3, PARP and CD4 staining) was performed to further evaluate the *in vivo* antitumor activity. As shown in Figure 6, the DOX-TNP and DOX groups displayed obvious nuclear condensation and fragmentation in the H & E images. In comparison, the cells in the vehicle and TNP groups retained normal morphologies. Consistent with Western blot analysis and IF assays, ABCB1 is overexpressed in SW620/Ad300 tumors in vehicle group, as compared to SW620 tumors. Interestingly, it was found that DOX-TNP and DOX can significantly upregulate the expression levels of ABCB1. As is well known, Caspase-3 and PARP are commonly used as biomarkers for cell apoptosis and CD4 is commonly used as a biomarker for cell necrosis [42–44]. As anticipated, the Caspase-3 and PARP expression levels were significantly increased in the DOX-TNP group, indicating that DOX-TNP caused the highest level of cell apoptosis in SW620 and SW620/Ad300 tumors, as compared to other three groups. Interestingly, CD4 staining suggested that the DOX-TNP group possessed the highest level of cell necrosis in SW620 and SW620/Ad300 tumors (Figure 6). Therefore, the IHC analyses are supportive of the potent anticancer activity of DOX-TNP.

## DISCUSSION

The evolution of chemotherapy for neoplastic diseases has advanced dramatically, from alkylating agents and antimetabolites to natural products, and latest molecular targeted drugs such as small molecule tyrosine kinase inhibitors and monoclonal antibodies. Antineoplastic drugs discovery endeavors are currently aimed at reducing the threshold for apoptosis in cancer cells, resensitizing cancer cells to drugs, meanwhile making their toxicities more manageable [45]. In this study, we established an ABCB1 overexpressing tumor xenograft mouse model to demonstrate that TNP is able to effectively deliver DOX into tumors and bypass the efflux function of ABCB1 transporter, thereby significantly increasing the intratumoral accumulation of DOX and potentiating the anticancer activity of DOX. First, MTT results suggested that DOX-TNP were more cytotoxic to CCD-18Co, SW620, and SW620/Ad300 cells than DOX. SW620 cells were more vulnerable to DOX-TNP than CCD-18Co, implying that DOX-TNP not only had improved anticancer efficacy, but also good anticancer selectivity. While the IC_50_ values of TNP were bigger than 300 μM for all three cell lines, suggesting that TNP is a safe, biocompatible nanocarrier (Table 1). Using the tumor xenograft mouse model, we discovered that TNP significantly strengthened the inhibitory effects of DOX on the growth of both SW620 and SW620/Ad300 tumors over an 18-day treatment period (Figures 2 and 3). Figure 2C showed that the tumor weights of SW620 tumors after the 18-day treatment of vehicle, TNP, DOX and DOX-TNP were 3.57 g, 3.54 g, 0.94 g, and 0.61 g. The tumor weights of SW620/Ad300 tumors after the 18-day treatment of vehicle, TNP, DOX and DOX-TNP were 2.38 g, 2.33 g, 1.33 g and 0.51 g, respectively (Figure 3C). By comparing the potency of anti-tumor effects between SW620 and SW620/Ad300 tumors, it was found that DOX and DOX-TNP can shrink SW620 tumors by 3.8 times and 5.9 times as compared to vehicle control group. Furthermore, DOX and DOX-TNP can inhibit the growth of SW620/Ad300 tumors by 1.8 times and 4.7 times. The only 1.8 times inhibition of DOX on the SW620/Ad300 tumors is much smaller than the 3.8 times shrinkage of SW620 tumors by DOX, revealing that the ABCB1-overexpressing SW620/Ad300 tumors are resistant to DOX treatment. Interestingly, the 4.7 times inhibition of DOX-TNP is obviously stronger than the 1.8 times inhibition of DOX on the SW620/Ad300 tumors. These findings indicate that DOX-TNP might have a better application in MDR tumors.

*In vitro* experiments are nonphysiological and have important limitations. DMEM culture medium is the solely environment for *in vitro* cancer cells. However, living creatures are biologically complex and this is especially true in higher order animals, including humans. Tumor environment *in vivo* is dependent on a more sophisticated system including circulatory system (extracellular and intracellular fluid), immune system, endocrine system, digestive system, etc. While the data from experiments carried out in cancer cells is important, *in vivo* studies, using animals, are necessary to investigate how drugs can produce therapeutic effects in a whole living organism. Many *in vivo* interactions are complex and cannot be obtained from *in vitro* experiments. Thus, using *in vitro* data as guidance and them performing *in vivo* experiments will be the best way to determine if DOX-TNP can be clinically useful in humans. Macrophages and other myeloid cells are ubiquitously distributed in solid tumor microenvironment and modulate tumor cytotoxicity by releasing various cytokines and other immune factors [46]. It has been reported that the levels of numerous cytokines (IL-6, TNF-α and G-CSF) as well as chemokines (RANTES, IP-10, MDC) were significantly increased after incubation of RAW 264.7 macrophages with the TNP for 24 h [38]. These findings suggest that DOX-TNP may produce stronger antitumor activity through their ability to modulate macrophage immune function, which can only be seen in the *in vivo* models.

Cardiomyopathy is the most dangerous side effect of DOX, leading to congestive heart failure [40]. The troponins include three proteins (troponin I, T and C) that regulate actin and myosin interactions during muscle contraction. Troponins T and I have distinct isoforms that exist in skeletal and cardiac muscle. The release of these proteins into the bloodstream from cardiomyocyte necrosis accounts for their application as biomarkers of cardiac muscle damage. It is reported that the increment of cTnI levels are associated with more severe cardiomyopathy [41]. Compared with DOX, the slight reduction of cTnI in DOX-TNP group was not statistically significant, indicating that TNP alone may not have cardiotoxicity effect in mice. Also cTnI levels in mice indicated that TNP had no cardiotoxicity and DOX-TNP had moderate cardiotoxicity. Myelosuppression is a major dose-limiting complication of DOX. Our results showed that the mean numbers of WBC and platelets in the four treatment groups were all in the normal range, suggesting that TNP and DOX-TNP did not cause neutropenia or thrombocytopenia in mice (Figure 4B and 4C). In a word, TNP is safe nanocarrier with excellent biocompatibility and minimal toxicity. As shown in the Figure 5D, the intratumoral concentration of DOX in DOX-TNP group was significantly higher than that in DOX group in both SW620 tumors (*P* < 0.05) and SW620/Ad300 tumors (*P* < 0.01), suggesting that TNP stimulated the delivery of DOX into tumors in the tumor microenvironment. In contrast, DOX-TNP did not have a significant effect on plasma concentrations of DOX (Figure 5C) 240 min after administration. Hence TNP-induced increment in the efficacy of DOX in SW620 and SW620/Ad300 tumors is partially due to its better drug delivery of DOX into tumors, thereby increasing the intratumoral accumulation of DOX.

*Ex vivo* IHC analysis of SW620 and SW620/Ad300 tumor sections is evidence of the prominent antitumor activity of DOX-TNP. Cells at the late stage of the apoptosis displayed morphological standards (chromatin condensation, DNA fragmentation and membrane blebbing) that could be readily identified by H & E staining [42]. In the H & E images, DOX-TNP and DOX group displayed obvious nuclear condensation and fragmentation (Figure 6). Interestingly, it was found that DOX-TNP and DOX can significantly upregulate the expression levels of ABCB1. Caspase-3 is a critical modulator of programmed cell death (apoptosis). PARP, a major substrate of caspases-3 and -7, is a useful marker of apoptosis [43]. The Caspase-3 and PARP staining indicated that the DOX-TNP group possessed the highest level of cell apoptosis in SW620 and SW620/Ad300 tumors, as compared to other three groups. Moreover, the CD4 staining displayed that the DOX-TNP group possessed the highest level of cell necrosis in SW620 and SW620/Ad300 tumors, as compared to other three groups (Figure 6).

DOX belongs to the group of anthracycline antibiotics and functions as a topoisomerase II inhibitor. When targeting topoisomerase II, DOX acts by stabilizing a reaction intermediate in which DNA strands are first cleaved and then covalently linked to certain tyrosine residues of the enzyme, eventually impeding DNA resealing. Hence the formation of DOX-DNA-topoisomerase II ternary complexes lead to the generation of irreversible double strand breaks and finally to the induction of cell death [47]. It has been reported that DOX induced dose-dependent G2/M and/or G1/S cell cycle arrest, followed by grade- and dose-dependent reduction in the amount of the cytosolic trimeric form of FasL, activation of Caspase-3, Caspase-8, Caspase-9, cleavage of PARP, Lamin A/C, Bcl-X_L/S_ and interestingly Hsp90, and finally cell death [48]. Table 1 showed the IC_50_ values of TNP were greater than 300 μM for all three cell lines, indicating that the TNP was nontoxic to cancer cells. Furthermore, our data demonstrated that TNP alone has no antitumor activity on SW620 and SW620/Ad300 tumors (Figures 2 and 3). Our data also indicated that TNP did not cause hematopoietic damage or cardiac toxicity (Figures 4 and 5B). Therefore, TNP potentiates the anticancer activity of DOX via facilitating the delivery of DOX into tumors and bypass the efflux function of ABCB1 transporter. DOX-TNP possessed the similar mechanism of DOX with increased potency.

In oncology, unique structural features of many solid tumors, including hypervasculature, defective vascular architecture, and impaired lymphatic drainage result in the well-characterized enhanced permeability and retention (EPR) effect, which is fundamental for nanotechnology platforms to deliver and accumulate drugs into tumors. Enhanced permeability of the tumor leaky vasculature allows macromolecules (DOX-TNP) to enter the tumor interstitial space and suppressed lymphatic filtration allows them to stay and accumulate there [49]. Moreover, the tumor microenvironment is different from plasma environment. For example, vasculature distribution, tumor blood flow (blood pressure) and composition of intratumoral fluid are different and can induce TNP to release more DOX from DOX-TNP into tumor tissues. These factors result in accumulation of nanoparticles in the tumor tissues. That's why TNP significantly enhanced DOX concentration in tumors but not in the plasma of mice.

Pegylated liposomal DOX is a very good example of DOX formulation with reduced cardiotoxicity and an improved pharmacokinetic profile. Chemotherapy with the anthracycline doxorubicin is a well established treatment approach in a number of malignancies; however, DOX is associated with adverse events that limit its usefulness, including the development of cumulative dose-related cardiotoxicity. The emergence of STEALTH^®^ technology has allowed the development of an enhanced DOX formulation; DOX has been encapsulated in liposomes with surface-bound methoxypolyethylene glycol (Doxil^®^) in a process known as pegylation, with the aim of retaining or improving the efficacy of the drug, while minimizing adverse events. Pegylation protects liposomes from detection by the mononuclear phagocyte system, thereby increasing the blood circulation time. Pegylated liposomal DOX is effective and tolerable in various malignancies, such as metastatic breast cancer, progressive ovarian cancer, relapsed or refractory multiple myeloma and AIDS-related Kaposi's sarcoma [50].

TNP potentiates the anticancer activity of DOX via facilitating the delivery of DOX into tumors and bypassing the efflux function of ABCB1 transporter. Furthermore, DOX-TNP may produce stronger antitumor activity through their ability to modulate macrophage immune function, such as by increasing the secretion of numerous cytokines (IL-6, TNF-α, and G-CSF) as well as chemokines (RANTES, IP-10, MDC) from macrophages. Consequently, the design of this infusion-dialysis based procedure for isolating organic nanoparticles from green tea and establishment of the drug-resistant tumor xenograft mouse model open up a new era in the development of natural nanomaterials for overcoming MDR in cancer cells and strengthening the anticancer activity of chemotherapeutic agents in cancer treatment.

## MATERIALS AND METHODS

### Chemicals and equipment

Laoshan^®^ green tea was purchased from China. (−) Epigallocatechin gallate (EGCG), caffeine, theobromine, 1,9-dimethyl-methylene blue (DMMB), chondroitin sulfate (CS), doxorubicin hydrochloride (DOX), HEPES, Sephadex G75 and phosphate buffered saline (PBS) were purchased from Sigma-Aldrich (St. Louis, MO). Dulbecco's modified Eagle's medium (DMEM), Eagle's Minimum Essential medium (EMEM), fetal bovine serum (FBS), penicillin/streptomycin and trypsin 0.25% were products of Hyclone, Thermo Scientific (Logan, UT). The monocolonal C219 (against ABCB1), monoclonal antibody BA3R (anti-beta actin) and the secondary horseradish peroxidase labeled rabbit anti-mouse IgG were purchased from Fisher Scientific. (Pittsburgh, PA). The monoclonal antibody P7965 (against ABCB1) was purchased from Sigma Chemical Co. (St. Louis, MO). The Alexa flour 488-conjugated goat anti-mouse IgG was purchased from Molecular Probes (Eugene, OR). Full-Range Rainbow Molecular Weight Marker was purchased from GE healthcare Life Sciences (Pittsburgh, PA). High sensitivity mouse cardiac troponin-I ELISA KIT was purchased from Life Diagnostics, Inc. (West Chester, PA). Paraformaldehyde, 3-(4,5-dimethylthiazol-2-yl)-2,5-diphenyl-tetrazolium bromide (MTT), dimethyl sulfoxide (DMSO) were obtained from Sigma Chemical Co. (St. Louis, MO). WBC Diluting Fluid and Platelet Diluent were purchased from Eng Scientific Inc. (Clifton, NJ). SAView^®^ (mouse/rabbit-HRP, DAB) IHC kit was obtained from Enzo Life Sciences, Inc. (Farmingdale, NY). A OPSYS microplate reader was purchased from Dynex Technologies (Chantilly, VA).

### Cell lines and cell culture

The human normal colon fibroblast cell line CCD-18Co was cultured at 37°C, 5% CO_2_, with ATCC-formulated Eagle's Minimum Essential Medium containing 10% FBS and 1% penicillin/streptomycin. The human colon cancer cell line SW620 and its doxorubicin-selected ABCB1 overexpressing subline SW620/Ad300 were cultured at 37°C, 5% CO_2_, with DMEM containing 10% FBS and 1% penicillin/streptomycin. All cells were grown as adherent monolayers in drug-free culture media for more than 2 weeks before the assay.

### Preparation of TNP

Green tea infusions were made by steeping 15 g of dried green tea leaves in 200 ml of boiling deionized water (DI water) for 20 min. The resulting hot tea infusion was then centrifuged at 5000 rpm for 10 min, and the supernatant was filtered through a 1 μm filter (Whatman Inc., Florham Park, NJ). The filtered solution was then sonicated in a water-bath sonicater (model 750D, VWR) at room temperature for 30 min. The free small molecules, such as alkaloids, and polyphenols, were removed by dialysis using 300KD MWCO tubing against DI water for 3 days at room temperature. Size-exclusion high-performance liquid chromatography (SEC-HPLC) was used to further isolate the nanoparticles after dialysis. 250 μl of the dialyzed solution was loaded onto a SEC-HPLC column (Phenomenex^®^BIOSEP-SEC-S4000), and eluted with distilled water at 1.0 ml/min of flow rate. The UV absorption at 280 nm was measured, and all fractions were collected.

### Preparation of DOX-TNP

The DOX-loaded TNP was prepared by mixing DOX (0.3 mM) with the TNP (1 mg/ml) in HEPES buffer (20 mM, pH = 7.0) at room temperature for 3 h. The DOX loaded into the TNP was isolated from the free DOX in the solution by a Sephadex G75 column method as previously reported [38].

### Cytotoxicity determination by MTT assay

The modified MTT colorimetric assay was used to detect the sensitivity of cells to anticancer drugs *in vitro* [51]. Cells were seeded in 180 μl of medium in 96-well plates in triplicate at 5000–6000 cells/well. After incubation at 37°C for 24 h, the cells were treated with different concentrations of chemotherapeutic drugs (20 μl/well). After 72 h of incubation at 37°C, 20 μl of MTT solution (4 mg/ml) was added to each well. The plates were further incubated at 37°C for 4 h, allowing viable cells to change the yellow-colored MTT into dark-blue formazan crystals. Subsequently, the MTT/medium was removed from each well without disturbing the cells, and 100 μl of DMSO was added into each well. Plates were placed on a shaking table to thoroughly mix the formazan into the solvent. Finally, the absorbance was determined at 570 nm by Opsys microplate reader (Dynex Technologies, Chantilly, VA).

### Western blot analysis

Equal amounts of total cell lysates (60 μg protein) and Full-Range Rainbow Molecular Weight Marker were resolved using 8–12% sodium dodecyl sulfate polyacrylamide gel electrophoresis (SDS-PAGE) and transferred onto polyvinylidene fluoride (PVDF) membranes through electrophoresis. The PVDF membranes were immersed in blocking solution (5% skim milk) in TBST buffer (10 mM Tris–HCl, pH 8.0, 150 mM NaCl, and 0.1% Tween 20) to block nonspecific binding for 1 h at room temperature. Then the PVDF membranes were immunoblotted overnight with primary monoclonal antibodies against either β-Actin at 1:200 dilution or ABCB1 at 1:200 dilution at 4°C, and were then further incubated for 2 h at room temperature with rabbit anti-mouse horseradish peroxide (HRP)-conjugated secondary antibody (1:2000 dilution). The protein–antibody complex was detected by an enhanced chemiluminescence detection system (Amersham, NJ). β-Actin was used to confirm equal loading in each lane in the samples prepared from cell lysates.

### Immunofluorescence (IF) assay

Cells (1 × 10^4^) were seeded in 96-well plate and harvested overnight. The cells were washed with PBS and fixed with 4% paraformaldehyde for 15 min at room temperature and then rinsed with PBS three times. Then cells were kept in 1% Triton X-100 for 10 min at 4°C and rinsed with PBS three times. Non-specific reactions were blocked with BSA (2 mg/ml) for 1 h at 37°C. The monoclonal antibody against ABCB1 (P7965) (1:200) (Sigma Chemical Co., St. Louis, MO) was applied overnight, followed by an Alexa flour 488-conjugated goat anti-mouse IgG (1:1000) (Molecular Probes, Eugene, OR) for 1 h. PI (Propidium iodide) was used to counterstain the nuclei. Images were taken with Nikon TE2000 inverted microscope (Nikon Instruments Inc. Melville, NY).

### Establishing the tumor xenograft mouse model

Male athymic NCR (nu/nu) nude mice (NCRNU M, homozygous, albino; 18–25 g; 6–10 week; Taconic Farms) were used for the tumor xenograft experiments. All animals were maintained on an alternating 12 hours light/dark cycle with ad libitum water and rodent chow. The SW620 and SW/Ad300 mouse models were designed for the first time with a slight modification to the KBv200 cell xenograft model previously established by Chen and colleagues [52, 53]. SW620 and SW/Ad300 (5.0 × 10^6^) cells were injected subcutaneously under the armpits. When the subcutaneous tumors reached approximately 5 × 5 mm in size, the mice were randomized into four treatment groups. There were 4 experimental groups with 8 mice in each group. Group 1 animals received the vehicle treatment. Group 2 animals received 0.71 mg/kg TNP. Group 3 animals received the anticancer drug 2.00 mg/kg DOX. Group 4 animals received DOX-TNP. All injections were prepared in 10 mM HEPES buffer and administered intra-peritoneally every 3 days. The tumor sizes were measured using calipers and body weights were recorded [54]. The body weights of the animals were monitored every 3 days to adjust the drug dosage and to determine the treatment-related toxicities as well as disease progression. The two perpendicular diameters of tumors were recorded every 3 days and tumor volumes were estimated [52, 54]. Blood plasma was collected at the end of experiments before euthanizing by carbon dioxide. Various organs and tumor tissue were excised and stored at −80°C. All mice were maintained at the St. John's University Animal Facility. This research was approved by the IACUC at St. John's University. All experiments were conducted in compliance with the Animal Welfare Act and other federal regulations. The animals were treated humanely and cared for in accordance with guidelines set forth by the American Association for Accreditation of Laboratory Animal Care and the U.S. Public Health Service Policy on Humane Care and Use of Laboratory Animals.

### Collection of plasma and tissues for pharmacokinetics study

In a separate group of experiments, mice bearing SW620 and SW620/Ad300 tumors were divided into two groups: (i) DOX; (ii) DOX-TNP. After treatment, the animals were anesthetized and blood was obtained using supraorbital punctures and placed in heparinized tubes. Plasma was harvested at 10, 30, 60, 120, or 240 min intervals after paclitaxel administration in both groups. In addition, the tumors and lungs were removed, weighed, snap-frozen in liquid nitrogen, and stored at −80°C until analysis [55].

### Extraction of doxorubicin from plasma and tissue homogenate samples

A simple, one-step protein precipitation with acetonitrile was used for sample preparation. Tumor tissues were homogenized in saline (1:2, v/v). Doxorubicin was extracted from plasma and tissue homogenate samples by precipitation with acetonitrile in 1:1 and 1:2 ratios (v/v), respectively. Samples were vortexed for 1 min, followed by centrifugation for 10 min at 10,000 rpm. The supernatant was transferred to insert vials, from which 20 ml was injected onto the high-performance liquid chromatography (HPLC) column. Samples with concentrations higher than the calibration range limit were appropriately diluted to fit within the working calibration curve.

### High-performance liquid chromatography conditions

To evaluate the concentration of DOX within the blood sample, the extracts were centrifuged at 12,000 rpm for 2 min and the supernatant was then loaded (100 μl per injection) onto a BioSep-SEC-s4000 column (300 mm × 7.8 mm, Phenomenex, Torrance, CA) that was mounted on an Agilent 1100 series HPLC system (Santa Clara, CA). The loaded sample was eluted with PBS, pH 7.4, at a flow rate of 1 ml/min, monitored with absorbance at 480 nm and 215 nm, simultaneously. The peak areas for the DOX fractions were recorded, and the concentration of DOX in the supernatant was quantitatively determined according to its calibration curve for peak area vs. concentration.

### Blood cell counting

Blood was drawn up to the 0.5 mark in a RBC pipette and diluted using Platelet Diluent. The sample was shaken for approximately 1 min. The fluid was expelled from the capillary end of the pipette. The chamber and coverslip were cleaned to ensure they are free of artifacts. The chamber was then charged and the cells were allowed to settle (15–20 min) before counting. Then, venous or capillary blood was drawn to the 0.5 mark in a WBC pipette and diluted using WBC Diluent. Again, the chamber was charged and the cells were allowed to settle before the number of WBCs in the four corners 1 mm^2^ area was counted.

### Mouse cardiac troponin-I ELISA assay

The plasma with heparin was prepared as quickly as possible after blood collection and stored at 4°C. The desired number of coated wells in the holder was secured and 100 μl of cTnl HRP Conjugate was dispensed into each well. Then 100 μl of standards and diluted samples were dispensed into appropriate wells and thoroughly mixed and incubated on an orbital shaker (150 rpm) at room temperature (18–25°C) for 60 min. Later, the incubation mixture was removed by flicking the plate contents into a waste container. The microtiter wells were washed and emptied 6 times with 1x wash solution. The entire wash procedure was performed as quickly as possible. After washing, the wells were struck sharply onto absorbent paper to remove all residual droplets. 100 μl of TMB reagent were dispensed into each well and incubated at room temperature for 20 min on an orbital shaker at ~150 rpm. The reaction was stopped by adding 100 μl Stop Solution to each well. The mixture was gently mixed and the absorbance was read at 450 nm with a microtiter well reader within 5 min of mixing.

### Immunohistochemistry (IHC) analysis

The deparaffinization was performed for the formalin-fixed, paraffin-embedded tissue sections. After that, the slides were placed in peroxidase quenching solution for 20–30 min and then placed in distilled water for 2 min. To retrieve the epitopes, 200 ml epitope retrieval buffer was placed in to a container and preheated. Slides were placed into the container steamed for 20–30 min. To block the non-specific reaction, tissue sections were circled with a hydrophobic barrier pen. Two drops (100 μl) of serum blocking solution were added to completely cover the tissue and the sections were incubated for 1 hour at room temperature. Later, the solution was drained and 2 drops (100 μl) of primary antibody were applied to completely cover the tissue and the sections were incubated at 4°C overnight.

The next day, the slides were rinsed with PBS 3 times with 2 min each. Two drops (100 μl) of secondary antibody were applied to completely cover the tissue and incubate at 37°C for 30 min. Then the slides were rinsed with PBS 3 times with 2 min each. For the enzyme conjugate, 2 drops (100 μl) of enzyme conjugate were applied to completely cover the tissue on each section and the sections were incubated at 37°C for 20 min. Then the slides were rinsed with PBS 3 times for 2 min each. For color development, the sections were incubated with DAB solution and developed 2–10 min under microscope control. The slides were washed with tap water and submerged in distilled water for 5 min. The slides were also submerged in Mayer's hematoxylin for 10–30 seconds for counterstaining. The slides were then washed with tap water for 5 min and submerged in distilled water for 5 min. Finally, dehydration, clearing and mounting were conducted.

### Statistical analysis

All experiments were repeated at least three times and the differences were determined by using the Student's *t*-test. The statistical significance was determined as *P* < 0.05.

## Abbreviations

TNP: tea nanoparticle
DOX: doxorubicin
DOX-TNP: doxorubicin-loaded tea nanoparticles
MDR: multidrug resistance
ABC: ATP-binding cassette
ABCB1: ABC transporter subfamily B member 1
GIST: gastrointestinal stromal tumor
NSCLC: non-small cell lung cancer
cTnI: cardiac troponin-I
IHC: immunohistochemistry

## References

[1] Jemal A, Murray T, Ward E, Samuels A, Tiwari RC, Ghafoor A, Feuer EJ, Thun MJ (2005). Cancer statistics, 2005. CA Cancer J Clin.

[2] Cockerill GS, Lackey KE (2002). Small molecule inhibitors of the class 1 receptor tyrosine kinase family. Curr Top Med Chem.

[3] Wang YJ, Zhang YK, Kathawala RJ, Chen ZS (2014). Repositioning of Tyrosine Kinase Inhibitors as Antagonists of ATP-Binding Cassette Transporters in Anticancer Drug Resistance. Cancers.

[4] Gottesman MM (2002). Mechanisms of cancer drug resistance. Annu Rev Med.

[5] Deeley RG, Westlake C, Cole SP (2006). Transmembrane transport of endo- and xenobiotics by mammalian ATP-binding cassette multidrug resistance proteins. Physiol Rev.

[6] Mao Q, Unadkat JD (2005). Role of the breast cancer resistance protein (ABCG2) in drug transport. AAPS J.

[7] Ambudkar SV, Kim IW, Xia D, Sauna ZE (2006). The A-loop, a novel conserved aromatic acid subdomain upstream of the Walker A motif in ABC transporters, is critical for ATP binding. FEBS Lett.

[8] Juliano RL, Ling V (1976). A surface glycoprotein modulating drug permeability in Chinese hamster ovary cell mutants. Biochim Biophys Acta.

[9] Schinkel AH, Mol CA, Wagenaar E, van Deemter L, Smit JJ, Borst P (1995). Multidrug resistance and the role of P-glycoprotein knockout mice. Eur J Cancer.

[10] Gottesman MM, Fojo T, Bates SE (2002). Multidrug resistance in cancer: role of ATP-dependent transporters. Nat Rev Cancer.

[11] Sarkadi B, Homolya L, Szakacs G, Varadi A (2006). Human multidrug resistance ABCB and ABCG transporters: participation in a chemoimmunity defense system. Physiol Rev.

[12] Roskoski R (2003). STI-571: an anticancer protein-tyrosine kinase inhibitor. Biochem Biophys Res Commun.

[13] Dean M, Annilo T (2005). Evolution of the ATP-binding cassette (ABC) transporter superfamily in vertebrates. Annu Rev Genomics Hum Genet.

[14] Sauna ZE, Smith MM, Muller M, Kerr KM, Ambudkar SV (2001). The mechanism of action of multidrug-resistance-linked P-glycoprotein. J Bioenerg Biomembr.

[15] Wang YJ, Kathawala RJ, Zhang YK, Patel A, Kumar P, Shukla S, Fung KL, Ambudkar SV, Talele TT, Chen ZS (2014). Motesanib (AMG706), a potent multikinase inhibitor, antagonizes multidrug resistance by inhibiting the efflux activity of the ABCB1. Biochem Pharmacol.

[16] Matsuo K, Eno ML, Ahn EH, Shahzad MM, Im DD, Rosenshein NB, Sood AK (2011). Multidrug resistance gene (MDR-1) and risk of brain metastasis in epithelial ovarian, fallopian tube, and peritoneal cancer. Am J Clin Oncol.

[17] Eechoute K, Sparreboom A, Burger H, Franke RM, Schiavon G, Verweij J, Loos WJ, Wiemer EA, Mathijssen RH (2011). Drug transporters and imatinib treatment: implications for clinical practice. Clin Cancer Res.

[18] Gao B, Russell A, Beesley J, Chen XQ, Healey S, Henderson M, Wong M, Emmanuel C, Galletta L, Johnatty SE, Bowtell D, Haber M, Australian Ovarian Cancer Study Group (2014). Paclitaxel sensitivity in relation to ABCB1 expression, efflux and single nucleotide polymorphisms in ovarian cancer. Sci Rep.

[19] Han JY, Lim HS, Yoo YK, Shin ES, Park YH, Lee SY, Lee JE, Lee DH, Kim HT, Lee JS (2007). Associations of ABCB1, ABCC2, and ABCG2 polymorphisms with irinotecan-pharmacokinetics and clinical outcome in patients with advanced non-small cell lung cancer. Cancer.

[20] Strable E, Johnson JE, Finn M (2004). Natural nanochemical building blocks: icosahedral virus particles organized by attached oligonucleotides. Nano letters.

[21] Zheng G, Chen J, Li H, Glickson JD (2005). Rerouting lipoprotein nanoparticles to selected alternate receptors for the targeted delivery of cancer diagnostic and therapeutic agents. Proc Natl Acad Sci U S A.

[22] Mulder WJ, Strijkers GJ, Van Tilborg GA, Cormode DP, Fayad ZA, Nicolay K (2009). Nanoparticulate assemblies of amphiphiles and diagnostically active materials for multimodality imaging. Acc Chem Res.

[23] Huang Y, Wang Y, Wang Y, Yi S, Fan Z, Sun L, Lin D, Anreddy N, Zhu H, Schmidt M (2015). Exploring naturally occurring ivy nanoparticles as an alternative biomaterial. Acta biomaterialia.

[24] Xia L, Lenaghan SC, Zhang M, Zhang Z, Li Q (2010). Naturally occurring nanoparticles from English ivy: an alternative to metal-based nanoparticles for UV protection. J Nanobiotechnology.

[25] Wang Y, Sun L, Yi S, Huang Y, Lenaghan SC, Zhang M (2013). Naturally occurring nanoparticles from arthrobotrys oligospora as a potential immunostimulatory and antitumor agent. Advanced Functional Materials.

[26] Zhang M, Liu M, Prest H, Fischer S (2008). Nanoparticles secreted from ivy rootlets for surface climbing. Nano letters.

[27] Favi PM, Yi S, Lenaghan SC, Xia L, Zhang M (2014). Inspiration from the natural world: from bio-adhesives to bio-inspired adhesives. J Adhes Sci Technol.

[28] Cormode DP, Jarzyna PA, Mulder WJ, Fayad ZA (2010). Modified natural nanoparticles as contrast agents for medical imaging. Adv Drug Deliv Rev.

[29] Jankun J, Selman SH, Swiercz R, Skrzypczak-Jankun E (1997). Why drinking green tea could prevent cancer. Nature.

[30] Paschka AG, Butler R, Young CY (1998). Induction of apoptosis in prostate cancer cell lines by the green tea component,(−)-epigallocatechin-3-gallate. Cancer letters.

[31] Negishi H, Xu JW, Ikeda K, Njelekela M, Nara Y, Yamori Y (2004). Black and green tea polyphenols attenuate blood pressure increases in stroke-prone spontaneously hypertensive rats. J Nutr.

[32] Chen H, Zhang M, Qu Z, Xie B (2008). Antioxidant activities of different fractions of polysaccharide conjugates from green tea (Camellia Sinensis). Food Chemistry.

[33] Fassina G, Buffa A, Benelli R, Varnier OE, Noonan DM, Albini A (2002). Polyphenolic antioxidant (−)-epigallocatechin-3-gallate from green tea as a candidate anti-HIV agent. Aids.

[34] Khan SG, Katiyar SK, Agarwal R, Mukhtar H (1992). Enhancement of antioxidant and phase II enzymes by oral feeding of green tea polyphenols in drinking water to SKH-1 hairless mice: possible role in cancer chemoprevention. Cancer Res.

[35] Chen C, Yu R, Owuor ED, Kong AT (2000). Activation of antioxidant-response element (ARE), mitogen-activated protein kinases (MAPKs) and caspases by major green tea polyphenol components during cell survival and death. Arch Pharm Res.

[36] Wynder EL, Rose DP, Cohen LA (1994). Nutrition and prostate cancer: a proposal for dietary intervention. Nutr Cancer.

[37] Pianetti S, Guo S, Kavanagh KT, Sonenshein GE (2002). Green tea polyphenol epigallocatechin-3 gallate inhibits Her-2/neu signaling, proliferation, and transformed phenotype of breast cancer cells. Cancer Res.

[38] Yi S, Wang Y, Huang Y, Xia L, Sun L, Lenaghan SC, Zhang M (2014). Tea Nanoparticles for Immunostimulation and Chemo-Drug Delivery in Cancer Treatment. J Biomed Nanotechnol.

[39] Nemzek J, Bolgos G, Williams B, Remick D (2001). Differences in normal values for murine white blood cell counts and other hematological parameters based on sampling site. Inflamm Res.

[40] Missov E, Calzolari C, Pau B (1997). Circulating cardiac troponin I in severe congestive heart failure. Circulation.

[41] Wang TJ (2007). Significance of circulating troponins in heart failure: if these walls could talk. Circulation.

[42] Bressenot A, Marchal S, Bezdetnaya L, Garrier J, Guillemin F, Plenat F (2009). Assessment of apoptosis by immunohistochemistry to active caspase-3, active caspase-7, or cleaved PARP in monolayer cells and spheroid and subcutaneous xenografts of human carcinoma. J Histochem Cytochem.

[43] Porter AG, Jänicke RU (1999). Emerging roles of caspase-3 in apoptosis. Cell Death Differ.

[44] Jin Z, El-Deiry WS (2005). Overview of cell death signaling pathways. Cancer Biol Ther.

[45] Zhang Y, Wang Y, Gupta P, Chen Z (2015). Multidrug Resistance Proteins (MRPs) and Cancer Therapy. AAPS J.

[46] Gajewski TF, Schreiber H, Fu Y (2013). Innate and adaptive immune cells in the tumor microenvironment. Nat Immunol.

[47] Kellner U, Sehested M, Jensen PB, Gieseler F, Rudolph P (2002). Culprit and victim–DNA topoisomerase II. Lancet Oncol.

[48] Stravopodis DJ, Karkoulis PK, Konstantakou EG, Melachroinou S, Lampidonis AD, Anastasiou D, Kachrilas S, Messini-Nikolaki N, Papassideri IS, Aravantinos G (2009). Grade-dependent effects on cell cycle progression and apoptosis in response to doxorubicin in human bladder cancer cell lines. Int J Oncol.

[49] Prabhakar U, Maeda H, Jain RK, Sevick-Muraca EM, Zamboni W, Farokhzad OC, Barry ST, Gabizon A, Grodzinski P, Blakey DC (2013). Challenges and key considerations of the enhanced permeability and retention effect for nanomedicine drug delivery in oncology. Cancer Res.

[50] Duggan ST, Keating GM (2011). Pegylated liposomal doxorubicin. Drugs.

[51] Carmichael J, DeGraff WG, Gazdar AF, Minna JD, Mitchell JB (1987). Evaluation of a tetrazolium-based semiautomated colorimetric assay: assessment of chemosensitivity testing. Cancer Res.

[52] Chen L, Liang Y, Ruan J, Ding Y, Wang X, Shi Z, Gu L, Yang X, Fu L (2004). Reversal of P - gp mediated multidrug resistance *in vitro* and *in vivo* by FG020318. J Pharm Pharmacol.

[53] Tiwari AK, Sodani K, Dai CL, Abuznait AH, Singh S, Xiao ZJ, Patel A, Talele TT, Fu L, Kaddoumi A, Gallo JM, Chen ZS (2013). Nilotinib potentiates anticancer drug sensitivity in murine ABCB1-, ABCG2-, and ABCC10-multidrug resistance xenograft models. Cancer letters.

[54] Dai CL, Tiwari AK, Wu CP, Su XD, Wang SR, Liu DG, Ashby CR, Huang Y, Robey RW, Liang YJ, Chen LM, Shi CJ, Ambudkar SV (2008). Lapatinib (Tykerb, GW572016) reverses multidrug resistance in cancer cells by inhibiting the activity of ATP-binding cassette subfamily B member 1 and G member 2. Cancer Res.

[55] Kathawala RJ, Wei L, Anreddy N, Chen K, Patel A, Alqahtani S, Zhang YK, Wang YJ, Sodani K, Kaddoumi A, Ashby CR, Chen ZS (2015). The small molecule tyrosine kinase inhibitor NVP-BHG712 antagonizes ABCC10-mediated paclitaxel resistance: a preclinical and pharmacokinetic study. Oncotarget.

